# HER2-positive is an independent indicator for predicting pathological complete response to neoadjuvant therapy and Ki67-changed after neoadjuvant chemotherapy predicts favorable prognosis in Chinese women with locally advanced breast cancer

**DOI:** 10.1097/MD.0000000000037170

**Published:** 2024-02-09

**Authors:** Yutong Fang, Qunchen Zhang, Yuan Wu, Jundong Wu

**Affiliations:** a The Breast Center, Cancer Hospital of Shantou University Medical College, Shantou, China; b The Department of Breast, Jiangmen Central Hospital, Jiangmen, China; c Department of Breast Surgery, Meizhou People’s Hospital, Meizhou, China.

**Keywords:** breast cancer, IHC markers, neoadjuvant chemotherapy, pathologic complete response, prognosis

## Abstract

The growing body of evidence suggests that breast cancer (BC) who achieve pathological complete response (pCR) after neoadjuvant chemotherapy (NAC) may experience a more favorable prognosis. The objective of this study is to investigate the correlation between clinicopathologic parameters of locally advanced breast cancer (LABC) patients and the outcomes of NAC, with the aim of identifying predictive indicators for pCR. Additionally, we seek to examine the conversion of IHC markers in pCR patients following NAC and its impact on the prognosis of BC patients. We conducted a study involving 126 patients with LABC. Clinicopathological parameters associated with pCR were subjected to univariate and multivariate analysis. Kaplan–Meier (KM) curves and the log-rank test were used to compare the statistical difference in prognosis in different groups of patients. Additionally, we used difference and consistency tests to examine the conversion of immunohistochemistry (IHC) markers following NAC. The status of estrogen receptor (ER), progesterone receptor (PR), human epidermal growth factor receptor 2 (HER2) and molecular subtypes of BC were associated with pCR in the univariate analysis (all *P* < .05), which may be potential markers to predict pCR. HER2 was identified as an independent factor for predicting pCR in the multivariate analysis. The pCR rate of HER2-positive patients who received NAC combined targeted therapy was higher than that of patients who only received NAC (*P* = .003). The disease-free survival (DFS) rate of TNBC patients who achieved pCR was significantly higher than that of non-pCR TNBC patients (*P* = .026). The IHC marker conversion after NAC mainly existed in PR (*P* = .041). Ki67 expression decreased in the luminal B subtype and increased in the HER2 enriched subtype after NAC (all *P* < .001). Patients with Ki67 expression change after NAC had longer overall survival (OS) and DFS than unchanged patients (all *P* < .05). HER2-positive is an independent indicator for predicting pCR, and HE2-positive patients who received NAC combined targeted therapy were favorable to achieving pCR. IHC markers of BC patients exhibit varying degrees of alterations after NAC, and changes in Ki67 expression after NAC could serve as a marker to predict a better prognosis.

## 1. Introduction

As the most common malignant neoplasms in females, breast cancer (BC) has been one of the most challenging conditions confronting the medical profession. In 2020, the global number of new BC cases was approximately 2.26 million, with approximately 0.42 million cases reported in China,^[1,2]^ accounting for nearly one-fifth of the global burden. Currently, treatments for BC still mainly rely on surgery, chemotherapy, radiotherapy, hormone therapy, and targeted therapy.^[3]^ Since Perou et al^[4]^ first divided BC into 4 subtypes according to transcriptional profiles in 2000, the molecular subtype of BC has gradually been considered as one of the crucial indicators for the selection of appropriate treatment strategies for BC as a supplement to traditional clinicopathologic parameters. Since Carey et al^[5]^ demonstrated that immunohistochemistry (IHC) can effectively substitute gene expression-based molecular subtyping, several IHC markers, particularly the human epidermal growth factor receptor 2 (HER2), estrogen receptor (ER), progesterone receptor (PR), and Ki67, have been extensively utilized for classifying the molecular subtype of BC in clinical settings, offering the advantages of convenience, affordability, high sensitivity, and high specificity. With the advancement of diagnosis and treatment means for BC, the 5-year survival rate for early-stage BC has improved to nearly 100%. However, the 5-year survival rate for metastatic BC is still no more than 30%.^[6]^ Thus, advanced BC remains a significant public health concern that poses a threat to the well-being of women globally.

Locally advanced breast cancer (LABC) refers to BC that has spread beyond the breast to nearby tissues or lymph nodes, but has not yet metastasized to distant organs. This stage of BC is often characterized by larger tumor size, involvement of nearby lymph nodes, and potential spread to surrounding tissues.^[7,8]^ Neoadjuvant Chemotherapy (NAC) has gradually been applied for the treatment of BC, especially LABC, which can downstage a locally advanced and inoperable tumor and convert it from inoperable to operable, and increase the opportunity of breast-conserving surgery.^[9,10]^ Research has demonstrated that the overall survival (OS) and disease-free survival (DFS) of BC patients who achieve pathological complete response (pCR) to NAC are significantly longer than those of non-pCR patients.^[11,12]^ With the development of molecular biology, more and more novel biomarkers are proven to be useful for predicting the effect of NAC and the prognosis of BC patients.^[13–15]^ Nevertheless, research on these emerging biomarkers is still in its early stages, and they have not been widely implemented in clinical settings. Their predictive accuracy requires further validation. Conversely, traditional clinicopathologic parameters are more readily accessible and widely available for detecting and predicting the impact of NAC and the prognosis of patients. Previous research has indicated that pCR after NAC is associated with the clinicopathologic parameters of BC patients^[16,17]^ thus in-depth studies of predictive indicators related to pCR are needed and crucial to developing a precise and individualized treatment plan for patients. In addition, previous studies have shown that NAC can change the expression of IHC markers after NAC,^[18,19]^ which might result in the conversion of the molecular subtype and prognosis of BC patients. Nevertheless, it remains unknown whether the conversion of IHC markers after NAC is linked to the prognosis of BC patients. Our study aimed to investigate the relationship between clinicopathologic parameters and the impact of NAC in order to identify indicators that can predict pCR in LABC patients. Additionally, we sought to examine the conversion of IHC markers in non-pCR patients after NAC and its potential impact on the prognosis. These findings may contribute to the selection of optimal treatment and further improvement of patient prognosis.

## 2. Materials and methods

### 2.1. Inclusion and exclusion criteria of patients

This study was approved by the Research Ethics Committee of the Cancer Hospital of Shantou University Medical College. Female BC patients who received NAC followed by surgical resection in the Breast Center of Cancer Hospital of Shantou University Medical College, from May 2014 to October 2018 were selected as our studied objects. The inclusion criteria of patients were as follows: patients aged from 18 to 75 with good physical condition and Eastern Cooperative Oncology Group score between 0 and 1; patients diagnosed with LABC by ultrasonography-guided core biopsies combined with imaging examinations and further underwent NAC followed by surgical resection; and patients with normal liver/kidney function and blood routine tests. Diagnosed with metastatic BC or severe organic disease that was unable to tolerate chemotherapy were deemed as exclusion criteria. In addition, patients who died not due to BC or gave up treatment were also excluded. All patients signed informed consent of NAC, and the use of their data for this study was informed.

### 2.2. Clinicopathological parameters, IHC and molecular subtypes

The tumor size and lymph node status were classified according to the seventh edition of the American Joint Committee on Cancer Cancer Staging Manual.^[20]^ Tumor markers included carcinoembryonic antigen (CEA) and carbohydrate antigen 153 (CA153) detection in the serum of LABC patients, among which CEA > 5 μg/L or CA153 > 35 U/mL was considered as high expression.

The detection and staining evaluation of IHC markers were conducted with biopsy tissues before NAC, and postoperative specimens after NAC. According to the recommendations for immunohistochemical hormone receptor (HR) testing proposed by the American Society of Clinical Oncology and College of American Pathologists,^[21]^ ER and PR staining in tumor cells ≥ 1% were considered positive, and < 1% were considered negative. HER2 staining in tumor cells was scored as 0+, 1+, 2+, and 3+, among which 0+ and 1+ were defined as negative, 3+ as positive, and if the score was 2+, additional fluorescence in situ hybridization (FISH) was further performed with a result that if the FISH testing was positive, HER2 was considered positive otherwise negative. Ki67 was defined as high expression if IHC staining ≥ 14% and low expression if < 14%. The classification basis for molecular subtypes was the 2013 St Gallen consensus guidelines^[22]^ that are shown in Table 1.

**Table 1:** Molecular subtypes of breast cancer according to the 2013 St Gallen consensus guidelines.

Molecular subtypes	Definition
Luminal A	ER (+), PR ≥ 20% (+), HER2 (−) and Ki-67 < 14%
Luminal B	ER (+) and HER2 (−), PR (−)/ < 20% (+) or Ki67 ≥ 14% (+); ER (+) and HER2 (+), any PR status and Ki67 expression
HER2 enriched	ER (−), PR (−) and HER2 (+)
TNBC	ER (−), PR (−) and HER2 (−)

ER = estrogen receptor, HER2 = human epidermal growth factor receptor 2, PR = progesterone receptor, TNBC = triple-negative breast cancer.

### 2.3. NAC regimens and curative effect evaluation

Specific drug doses of chemotherapy or targeted drugs calculated according to the patient’s body surface area or weight, as well as main NAC regimens in this research are listed in Table 2. All chemotherapy drugs were injected intravenously through an implanted infusion port in patients on the first day of every 21-day cycle.

**Table 2:** Main chemotherapy regimens, dose and cycle of chemotherapy drugs.

Chemotherapy regimens	Dose and cycle of chemotherapy drugs		
EC-T	Epirubicin	85–100 mg/m^2^	4 cycles
	Cyclophosphamide	600 mg/m^2^	
	Docetaxel (followed)	85–100 mg/m^2^	4 cycles
EC-TH	Epirubicin	85–100 mg/m^2^	4 cycles
	Cyclophosphamide	600 mg/m^2^	
	Docetaxel (followed)	85–100 mg/m^2^	4 cycles
	Trastuzumab (followed)	6 mg/kg (8 mg/kg for first dose)	
FEC-T	5-fluorouracil	500 mg/m^2^	3 cycles
	Epirubicin	85–100 mg/m^2^	
	Cyclophosphamide	500 mg/m^2^	
	Docetaxel (followed)	85–100 mg/m^2^	3 cycles
TEC	Docetaxel	75 mg/m^2^	6 cycles
	Epirubicin	500 mg/m^2^	
	Cyclophosphamide	75 mg/m^2^	

EC-T = epirubicin plus cyclophosphamide followed by docetaxel, EC-TH = epirubicin plus cyclophosphamide followed by docetaxel and trastuzumab, FEC-T = 5-fluorouracil, epirubicin and cyclophosphamide followed by docetaxel, TEC = docetaxel, epirubicin and cyclophosphamide.

Patients after each cycle of NAC were informed to reexamine blood routine and liver/kidney function tests to timely detect the occurrence of some severe chemotherapy-related complications, especially myelosuppression, liver injury, and kidney injury for further treatment. Before each cycle of NAC, patients were informed to receive a physical examination, liver/kidney function tests, blood routine tests, serum tumor marker tests, electrocardiogram, and imaging examinations to observe tumor size change and confirm their physical conditions that could continue receiving the current treatment regimens. Tumor response was evaluated by RECIST version 1.1^[23]^ every 2 NAC cycles, and NAC regimens of patients whose response was progressive disease (PD) or stable disease (SD) were changed in time.

Surgery was conducted after 2 weeks of the last cycle of NAC treatment, and the surgical specimens were assessed blindly by 2 pathologists. Response to NAC after all treatment cycles were assessed according to the Miller-Payer system^[24]^ and the estimated reduction of tumor cells between Grade 2 to Grade 4 was considered as pathological partial response (pRR) and Grade 5 as pCR.

### 2.4. Statistical analysis

Statistical analysis of data was conducted by SPSS version 23. Receiver operating characteristic (ROC) analysis was performed to evaluate the clinicopathological parameters’ predictive power of response to NAC. Univariate analysis used the chi-square test, and multivariate analysis used the binary logistic regression analysis to analyze the relationship between the clinicopathological parameters and the response to NAC. Kaplan–Meier survival analysis and the log-rank test were used to compare the statistical difference of OS and DFS in different groups of patients. DFS was defined as from the time of surgery to the time of local recurrence or distant metastasis, and OS was defined as from the time of confirmed diagnosis to the time of death due to any cause. McNemar matching chi-square test and Kappa consistency test were respectively used to compare the difference and consistency in ER, PR and HER2 status before and conversion after NAC. Paired-samples *t* test and Spearman correlation analysis were used to evaluate the difference and consistency of Ki67 expression change before and after NAC. *P* values < .05 were considered statistically significant.

## 3. Result

### 3.1. Clinicopathological parameters and NAC regimens

See Table 3 for details, a total of 126 LABC female patients with a median age of 51 (ranging from 29 to 71) were selected as our studied objects. Among the 126 patients, 2 HER2 scored 2 + patients did not further receive the FISH test. From the result, we observed that more than half of the patients (54.0%) were postmenopausal, and 15.1% were younger than 40 years when initially diagnosed. Noteworthy, only 20.6% of patients had high expression levels of serum CEA or CA153 when diagnosed with BC.

**Table 3:** Clinicopathological parameters of breast cancer patients.

Clinicopathological parameters		N	Percentage (%)
Age (yr)	≤40	19	15.1
	>40	107	84.9
Menstrual status	Premenopausal	58	46.0
	Postmenopausal	68	54.0
Diabetes mellitus	No	103	81.7
	Yes	23	18.3
Tumor markers	Normal	100	79.4
	High expression	26	20.6
Tumor size	T1	3	2.4
	T2	28	22.2
	T3	34	27.0
	T4	61	48.4
Lymph node status	N0	13	10.3
	N1	36	28.6
	N2	49	38.9
	N3	28	22.2
Pathological classification	Ductal carcinoma	121	96.0
	Lobular carcinoma	1	0.8
	Other	4	3.2
ER	Positive	76	60.3
	Negative	50	39.7
PR	Positive	55	43.7
	Negative	71	56.3
HER2	Positive	51	40.5
	Negative	73	57.9
	Indeterminate	2	1.6
Ki67	<14	12	9.5
	≥14	114	90.5
Clinical stage	IIA	1	0.8
	IIB	3	2.4
	IIIA	50	39.7
	IIIB	44	34.9
	IIIC	28	22.2
Molecular subtypes	Luminal A	6	4.8
	Luminal B	70	55.6
	HER2 enriched	25	19.8
	TNBC	23	18.3

ER = estrogen receptor, HER2 = human epidermal growth factor receptor 2, PR = progesterone receptor, TNBC = triple-negative breast cancer.

The main NAC regimens were anthracyclines combined with paclitaxel (93.7%). Of the 51 HER2-positive patients, 37 (72.5%) only received NAC without targeted therapy due to economic burden. Most patients (93%) in this research had completed all treatment cycles of initial scheduled NAC regimens. Due to partial patients dying of BC before completing NAC and partially having to change NAC regimens because of PD or SD, we did not select NAC regimens as an indicator for further analysis.

### 3.2. HER2 was an independent indicator for predicting primary tumor pCR

**Figure 1.**
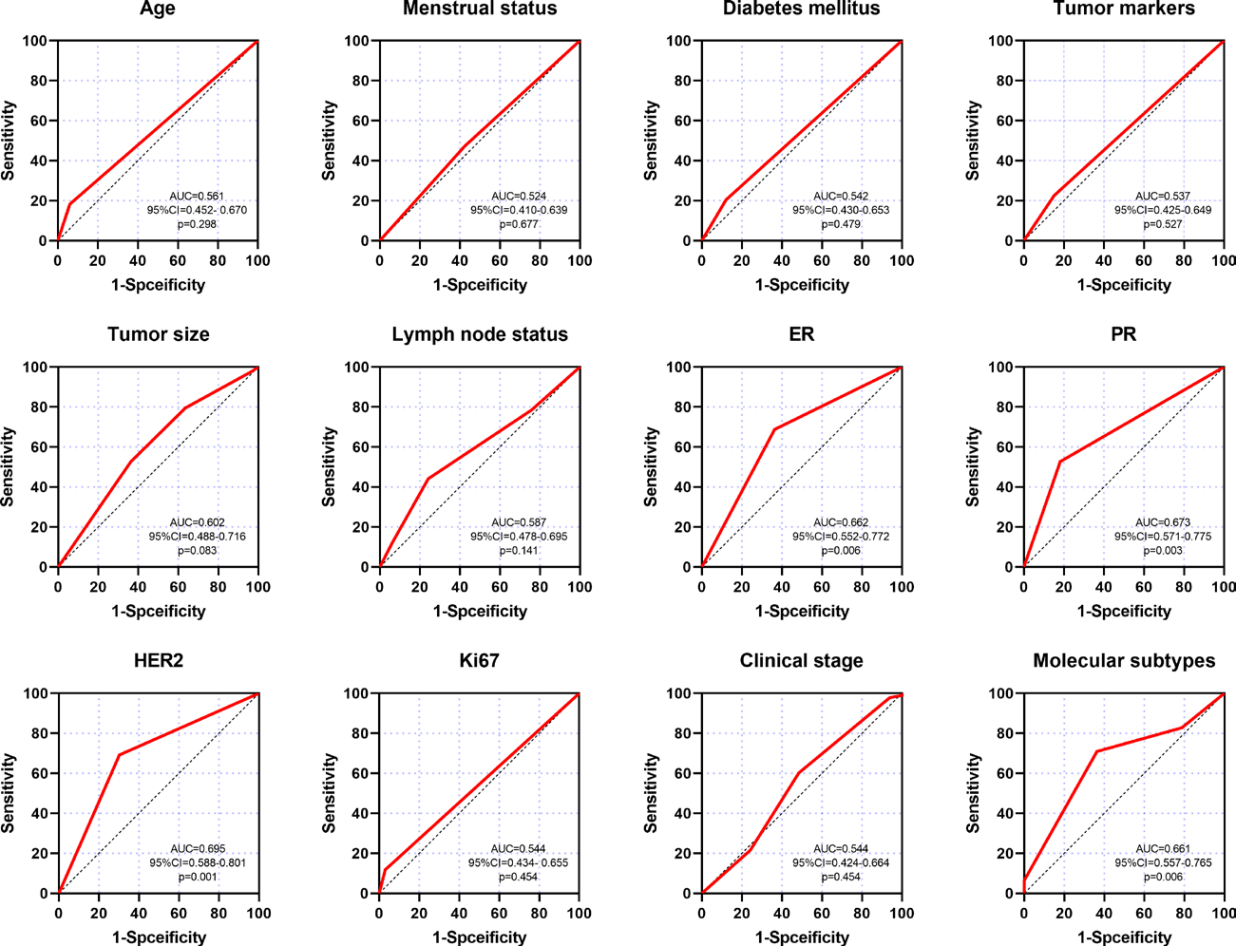
ROC analysis to evaluate the clinicopathological parameters’ predictive power of response to NAC. NAC = Neoadjuvant Chemotherapy.

Among the 126 patients, 33 achieved pCR, and the primary tumor pCR rate was 26.19%. Detailed results of the univariate analysis of the relationship between clinicopathological parameters and primary tumor pCR are shown in Table 4, a total of 4 clinicopathological parameters significantly associated with pCR were ER, PR, HER2, and molecular subtypes of BC (*P* < .05). In our cohort, the pCR rate of ER-negative patients was 42.0%, significantly higher than that of ER-positive patients (15.8%) (*P* = .001). The pCR rate of PR-negative patients (38.0%) was also significantly higher than that of positive patients (10.9%) (*P* = .001). In addition, the positive status of HER2 was associated with a higher pCR rate (45.1%) (*P* < .001), 31.4% higher than that of HER2-negative patients (13.7%). The above results revealed that HER2 enriched BC patients were more likely to receive better therapeutic effects from NAC than other molecular subtypes of BC patients. Further analysis indicated that the pCR rate of HER2 enriched BC patients was highest (56.0%), followed by which was triple-negative BC (TNBC) (30.4%), both significantly higher than that of luminal B (16.7%) and luminal A (0.0%) BC patients (*P* = .001). ROC analysis also revealed that ER (AUC = 0.662, *P* = .006), PR (AUC = 0.673, *P* = .003), HER2 (AUC = 0.695, *P* = .001), and molecular subtypes (AUC = 0.661, *P* = .006) may potential markers to predict pCR for LABC patients (Fig. 1).

**Table 4:** Relationship between clinicopathological parameters and primary tumor pCR in univariate analysis.

Clinicopathological parameters		Response to NAC of tumor			
		non-pCR (n = 93)	pCR (n = 33)	χ^2^	*P*
Age (yr)	≤40	17 (18.3%)	2 (6.1%)	2.840	.092
	>40	76 (81.7%)	31 (93.9%)		
Menstrual status	Premenopausal	44 (47.3%)	14 (42.4%)	0.234	.628
	Postmenopausal	49 (52.7%)	19 (57.6%)		
Diabetes mellitus	No	74 (79.6%)	29 (87.9%)	1.127	.288
	Yes	19 (20.4%)	4 (12.1%)		
Tumor markers	Normal	72 (77.4%)	28 (84.8%)	0.821	.365
	High expression	21 (22.6%)	5 (15.2%)		
Tumor size	T1	2 (2.1%)	1 (3.0%)	3.905	.272
	T2	17 (18.3%)	11 (33.3%)		
	T3	25 (26.9%)	9 (27.3%)		
	T4	49 (52.7%)	12 (36.4%)		
Lymph node status	N0	11 (11.8%)	2 (6.1%)	4.389	.222
	N1	30 (32.3%)	6 (18.2%)		
	N2	32 (34.4%)	17 (51.5%)		
	N3	20 (21.5%)	8 (24.2%)		
ER	Positive	64 (68.8%)	12 (36.4%)	10.718	.001
	Negative	29 (31.2%)	21 (63.3%)		
PR	Positive	49 (52.7%)	6 (18.2%)	11.790	<.001
	Negative	44 (47.3%)	27 (81.8%)		
HER2	Positive	28 (30.8%)	23 (69.7%)	15.157	<.001
	Negative	63 (69.2%)	10 (30.3%)		
Ki67	<14	11 (11.8%)	1 (3.0%)	2.188	.139
	≥14	82 (88.2%)	32 (97.0%)		
Clinical stage	IIB	2 (2.2%)	2 (6.1%)	3.090	.378
	IIIA	35 (37.6%)	15 (45.5%)		
	IIIB	36 (38.7%)	8 (24.2%)		
	IIIC	20 (21.5%)	8 (24.2%)		
Molecular subtypes	Luminal A	6 (6.5%)	0 (0.0%)	17.214	<.001
	Luminal B	60 (64.5%)	12 (36.4%)		
	HER2 enriched	11 (11.8%)	14 (42.4%)		
	TNBC	16 (17.2%)	7 (21.2%)		

ER = estrogen receptor, HER2 = human epidermal growth factor receptor 2, NAC = neoadjuvant chemotherapy, pCR = pathological complete response, PR = progesterone receptor, TNBC = triple-negative breast cancer.

If those HER2 enriched BC patients receive a better therapeutic effect from NAC was associated with targeted therapy? As shown in Table 5, a total of 14 patients received targeted therapy, accounting for 27.5% of HER2-positive BC patients. The result of the analysis revealed that the group of patients who received targeted therapy combined with NAC had a significantly higher pCR rate (78.6%) than the only NAC treatment group (32.4%) (*P* = .003). Thus the therapeutic effect of NAC combined with trastuzumab was better relative to only received NAC for HER2-positive patients.

**Table 5:** Relationship between treatment regimens and tumor pCR in HER2-positive patients.

Response to NAC of tumor	Treatment regimens			
	NAC combined targeted therapy (n = 14)	NAC (n = 37)	χ^2^	*P*
pCR	11 (78.6%)	12 (32.4%)	8.733	.003
non-pCR	3 (21.4%)	25 (67.6%)		

HER2 = human epidermal growth factor receptor 2, NAC = neoadjuvant chemotherapy, pCR = pathological complete response.

In addition, ER PR and HER2 were included for multivariate analysis, and molecular subtypes was excluded due to collinearity. The result shown in Table 6 revealed that HER2 was an independent indicator for predicting response to NAC, and that patients with positive HER2 status were more likely to achieve pCR after NAC.

**Table 6:** Relationship between clinicopathological parameters and primary tumor pCR in multivariate analysis.

Clinicopathological parameters	Coefficient	SE	*P*	OR	95% CI
Constant	−1.084	0.414	.009	0.338	0.143–0.735
ER (negative vs positive)	−0.689	0.534	.197	0.502	0.17–1.411
PR (negative vs positive)	−0.838	0.617	.175	0.433	0.124–1.455
HER2 (negative vs positive)	1.398	0.461	.002	4.046	1.671–10.308

ER = estrogen receptor, HER2 = human epidermal growth factor receptor 2, pCR = pathological complete response, PR = progesterone receptor.

### 3.3. Prognosis of primary tumor pCR and non-pCR patients

**Figure 2.**
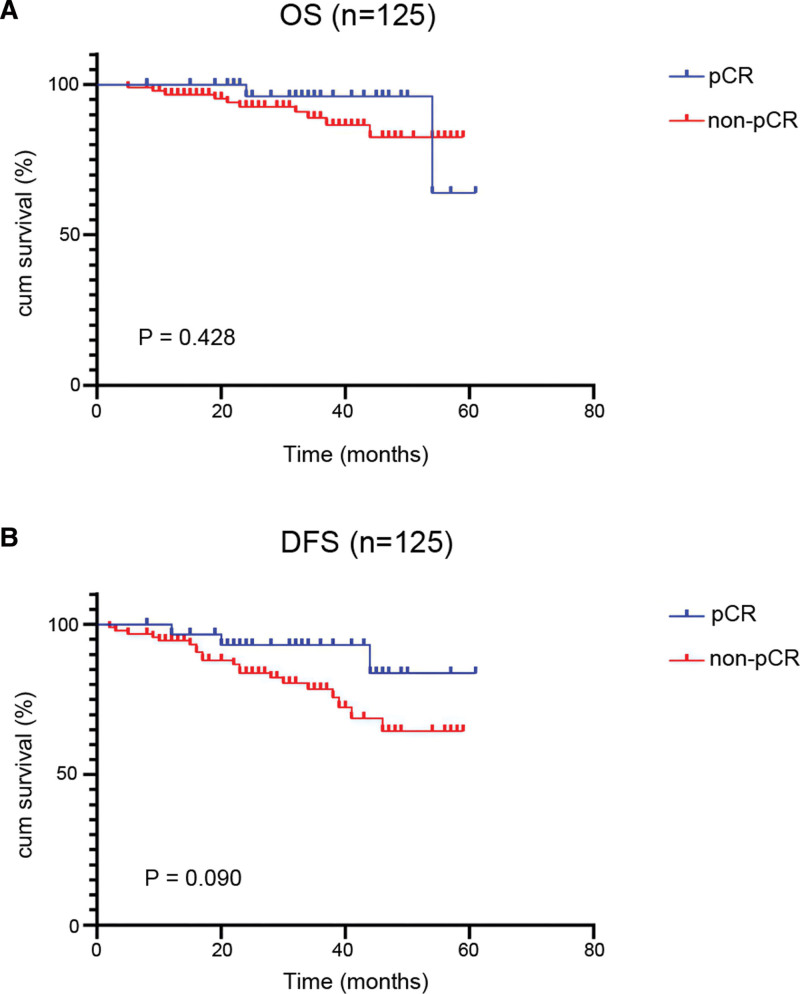
KM survival curves for OS (A) and DFS (B) of pCR and non-pCR patients. DFS = disease-free survival, KM = Kaplan-Meier, OS = overall survival, pCR = pathological complete response.

Among the 126 patients, one had been lost to follow-up after surgical resection, with a rate of loss to follow-up of 0.8%. The follow-up time ranged from 5 to 61 months, with a median follow-up time of 34 months. Of the 125 follow-up patients, 32 achieved pCR, among which relapse occurred in 3 patients and 2 died of a tumor. Among the 93 non-pCR patients, 20 cases had a local relapse or distant metastasis, and 10 died due to a tumor. The OS and DFS rates of 32 pCR patients were 89.2% and 78.5%, respectively. And the OS and DFS rates of the non-pCR group were respectively 89.2% and 78.5%, respectively 4.6% and 12.1% lower than the pCR group, exhibiting a tendency that pCR patients would have a relatively better prognosis even though without statistical significance (*P* = .428; *P* = .090, respectively) (Fig. 2).

**Figure 3.**
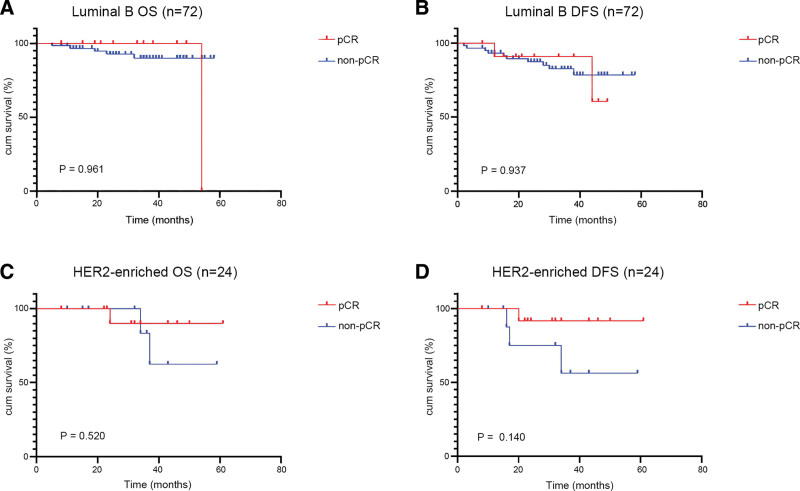
KM survival curves for OS and DFS of pCR and non-pCR luminal B (A, B) and HER2 enriched (C, D) patients. DFS = disease-free survival, HER2 = human epidermal growth factor receptor 2, KM = Kaplan–Meier, OS = overall survival, pCR = pathological complete response.

**Figure 4.**
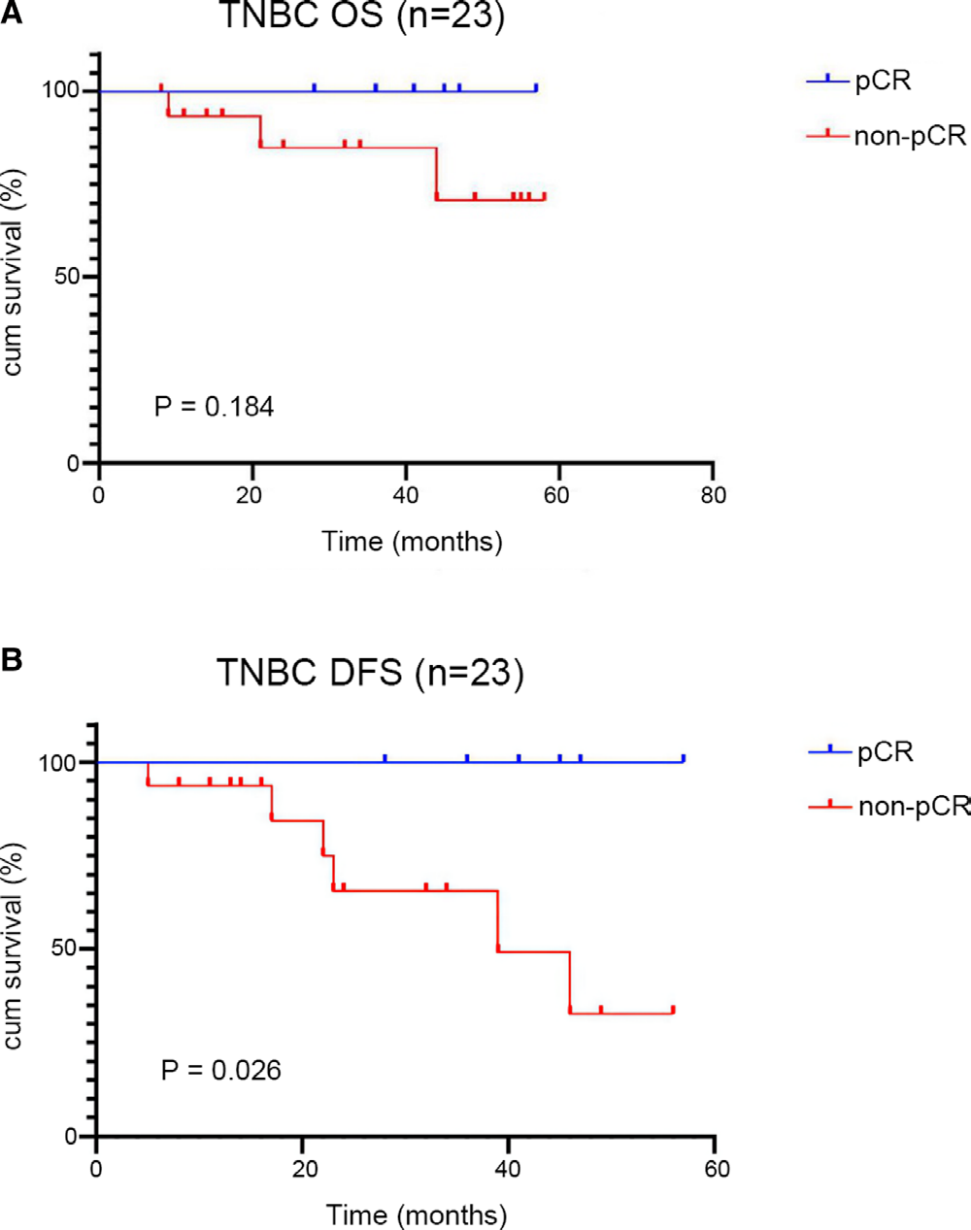
KM survival curves for OS (A) and DFS (B) of pCR and non-pCR TNBC patients. DFS = disease-free survival, KM = Kaplan–Meier, OS = overall survival, pCR = pathological complete response, TNBC = triple-negative BC.

We further compared the OS and DFS rates in various molecular subtypes of BC, and 6 patients diagnosed with luminal A BC were all non-pCR patients and thus did not be analyzed. Of the 72 luminal B cases, the OS and DFS rates of the pCR group were 91.7% and 83.3%, respectively higher than that of the non-pCR group, with OS and DFS rates of 89.2% and 78.5% respectively, but without statistically significant difference (*P* = .961; *P* = .937, respectively) (Fig. 3). The OS and DFS rates of 24 HER2-enriched patients were both 92.3% in the pCR group, and the OS and DFS rates of the non-pCR group were respectively 81.8% and 72.7% (*P* = .520; *P* = .140, respectively) (Fig. 3). Of the 23 TNBC patients, the DFS rate (100%) of the pCR group was significantly higher than that (62.5%) of the non-pCR group (*P* = .026) (Fig. 4). In addition, the OS rate (100.0%) of pCR TNBC patients was higher than that (81.3%) of non-pCR TNBC patients even though without a statistical difference (*P* = .184) (Fig. 4).

### 3.4. Prognosis of primary tumor and/or axillary lymph nodes (ALN) pCR/non-pCR patients

**Figure 5.**
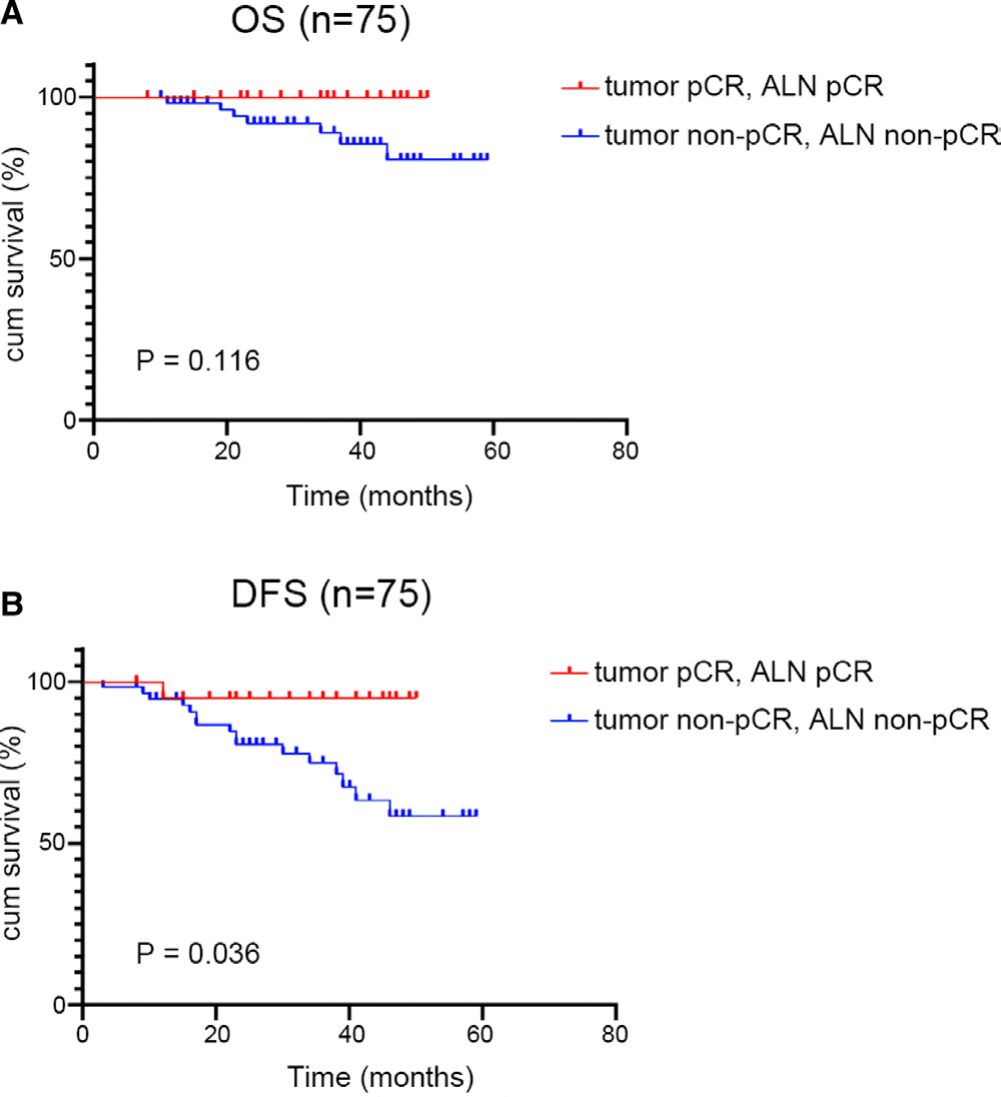
KM survival curves for OS (A) and DFS (B) of both tumor and ALN pCR and both tumor and ALN non-pCR patients. DFS = disease-free survival, KM = Kaplan-Meier, OS = overall survival, pCR = pathological complete response.

After the exclusion of 1 patient who was lost to follow-up, a total of 112 patients with ALN metastasis. As shown in Table 7, both the OS and DFS rates of the group that both primary breast tumor and ALN achieved pCR were higher than other groups. It is noteworthy that Kaplan–Meier survival analysis suggested that the DFS of the both tumor and ALN pCR group was significantly longer than that of both tumor and ALN non-pCR group (*P* = .036), and the OS exhibited the same tendency. (*P* = .116) (Fig. 5).

**Table 7:** OS and DFS of primary tumor and/or ALN pCR/non-pCR patients.

Response to NAC of primary tumor and ALN	OS rate	DFS rate
Primary tumor and ALN pCR	100.0%	95.5%
Primary tumor pCR, ALN non-pCR	87.5%	87.5%
Primary tumor non-pCR, ALN pCR	86.7%	86.2%
Primary tumor and ALN non-pCR	88.7%	71.7%

ALN = axillary lymph nodes, DFS = disease-free survival, NAC = neoadjuvant chemotherapy, OS = overall survival, pCR = pathological complete response.

### 3.5. Conversion of ER, PR, HER2, and molecular subtypes of non-pCR patients

After the exclusion of 2 patients who did not further receive the FISH test with a HER2 score of 2+, the conversion of IHC markers and molecular subtypes of a total of 91 non-pCR patients were observed. The conversion rates of IHC markers ER, PR, and HER2 from positive status before NAC to negative status after NAC were 2.2%, 16.5%, and 1.1%, respectively. With regard to changes from negative status to positive status, conversion rates of ER, PR, and HER2 were 2.2%, 5.5%, and 5.5%, respectively. The difference analysis of ER, PR, and HER2 are depicted in Table 8. The conversion mainly existed in PR (*P* = .041), among which 22.4% (13/58) cases of luminal B BC changed from positive to negative after NAC (*P* = .021). The consistency analysis shown in Table 9 revealed that ER (Kapper = 0.899, *P* < .001) and HER2 (Kapper = 0.851, *P* < .001) show good agreement before and after NCA, and PR (Kapper = 0.563, *P* < .001) show general agreement.

**Table 8:** McNemar matching chi-square test of IHC markers before and after NAC of non-pCR patients.

Molecular subtype	IHC marker	N	Conversion of IHC markers after NAC				*P*
			(+) to (+)	(+) to (−)	(−) to (+)	(−) to (−)	
Total	ER	91	60 (65.9%)	2 (2.2%)	2 (2.2%)	27 (29.7%)	>.999
	PR		32 (35.2%)	15 (16.5%)	5 (5.5%)	39 (42.9%)	.041
	HER2		27 (30.0%)	1 (1.1%)	5 (5.5%)	58 (63.7%)	.219
Luminal A	ER	6	6 (100.0%)	0 (0.0%)	0 (0.0%)	0 (0.0%)	–
	PR		4 (66.7%)	2 (33.3%)	0 (0.0%)	0 (0.0%)	–
	HER2		0 (0.0%)	0 (0.0%)	0 (0.0%)	6 (0.0%)	–
Luminal B	ER	58	54 (93.1%)	2 (3.4%)	1 (1.7%)	1 (1.7%)	>.999
	PR		28 (48.3%)	13 (22.4%)	3 (5.2%)	14 (24.1%)	.021
	HER2		16 (27.6%)	1 (1.7%)	4 (6.90%)	37 (63.8%)	.375
HER2 enriched	ER	11	0 (0.0%)	0 (0.0%)	1 (9.1%)	10 (90.9%)	–
	PR		0 (0.0%)	0 (0.0%)	0 (0.0%)	11 (100.0%)	–
	HER2		11 (100.0%)	0 (0.0%)	0 (0.0%)	0 (0.0%)	–
TNBC	ER	16	0 (0.0%)	0 (0.0%)	0 (0.0%)	16 (100.0%)	–
	PR		0 (0.0%)	0 (0.0%)	2 (12.5%)	14 (87.5%)	–
	HER2		0 (0.0%)	0 (0.0%)	1 (6.3%)	15 (93.8%)	–

HER2 = human epidermal growth factor receptor 2, IHC = immunohistochemistry, NAC = neoadjuvant chemotherapy, pCR = pathological complete response, TNBC = triple-negative breast cancer.

**Table 9:** Kappa Consistency test of IHC markers before and after NAC of non-pCR patients

Molecular subtype	IHC marker	N	Conversion of IHC markers after NAC				Kappa	*P*
			(+) to (+)	(+) to (−)	(−) to (+)	(−) to (−)		
Total	ER	91	60 (65.9%)	2 (2.2%)	2 (2.2%)	27 (29.7%)	0.899	<.001
	PR		32 (35.2%)	15 (16.5%)	5 (5.5%)	39 (42.9%)	0.563	<.001
	HER2		27 (30.0%)	1 (1.1%)	5 (5.5%)	58 (63.7%)	0.851	<.001
Luminal A	ER	6	6 (100.0%)	0 (0.0%)	0 (0.0%)	0 (0.0%)	–	–
	PR		4 (66.7%)	2 (33.3%)	0 (0.0%)	0 (0.0%)	–	–
	HER2		0 (0.0%)	0 (0.0%)	0 (0.0%)	6 (0.0%)	–	–
Luminal B	ER	58	54 (93.1%)	2 (3.4%)	1 (1.7%)	1 (1.7%)	0.374	.004
	PR		28 (48.3%)	13 (22.4%)	3 (5.2%)	14 (24.1%)	0.432	<.001
	HER2		16 (27.6%)	1 (1.7%)	4 (6.90%)	37 (63.8%)	0.802	<.001
HER2 enriched	ER	11	0 (0.0%)	0 (0.0%)	1 (9.1%)	10 (90.9%)	–	–
	PR		0 (0.0%)	0 (0.0%)	0 (0.0%)	11 (100.0%)	–	–
	HER2		11 (100.0%)	0 (0.0%)	0 (0.0%)	0 (0.0%)	–	–
TNBC	ER	16	0 (0.0%)	0 (0.0%)	0 (0.0%)	16 (100.0%)	–	–
	PR		0 (0.0%)	0 (0.0%)	2 (12.5%)	14 (87.5%)	–	–
	HER2		0 (0.0%)	0 (0.0%)	1 (6.3%)	15 (93.8%)	–	–

HER2 = human epidermal growth factor receptor 2, IHC = immunohistochemistry, NAC = neoadjuvant chemotherapy, pCR = pathological complete response, TNBC = triple-negative breast cancer.

Of the 91 non-pCR patients, 16 cases (17.6%) had molecular subtypes conversion after NAC. As shown in Table 10, 67% of luminal A cases converted to luminal B after NAC. 9% luminal B converted to luminal A, 3% to HER2-enriched, and 2% to TNBC. There was 9% HER2-enriched converted to luminal B subtype, 13% TNBC converted to HER2-enriched subtype, also 6% TNBC to luminal B subtype.

**Table 10:** Molecular subtypes before and after NAC of non-pCR patients.

Molecular subtypes before NAC	Molecular subtypes after NAC				
	Luminal A	Luminal B	HER2 enriched	TNBC	Total
Luminal A	2	4	–	–	6
Luminal B	5	50	2	1	58
HER2 enriched	–	1	10	–	11
TNBC	–	2	1	13	16
Total	7	57	13	14	91

HER2 = human epidermal growth factor receptor 2, NAC = neoadjuvant chemotherapy, pCR = pathological complete response, TNBC = triple-negative breast cancer.

### 3.6. Conversion of Ki67 after NAC was associated with prognosis of non-pCR patients

**Figure 6.**
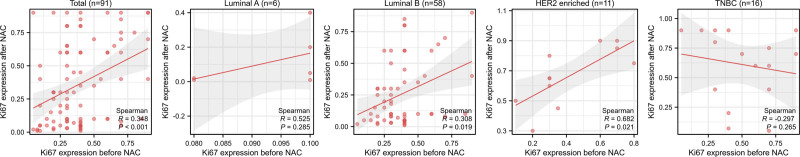
Spearman correlation analysis of Ki67 expression before and after NAC.

With regard to the detailed conversion of Ki67, see Table 11. In luminal A BC, the conversation of Ki67 was not evident (*P* = .742), and Ki67 expression levels significantly reduced after NAC in the luminal B subtype (*P* < .001). In HER2-enriched BC, Ki67 expression remarkably increased (*P* < .001), and there was also an increased tendency in TNBC (*P* = .323). Spearman correlation analysis showed that Ki67 (*R* = 0.348, *P* < .001) exhibits general agreement before and after NAC (Fig. 6).

**Table 11:** Paired-samples *t* test of Ki67 before and after NAC of non-pCR patients.

Molecular subtypes	N	Ki67 expression (%)		*P*
		Before NAC	After NAC	
Total	91	38.56 ± 21.53	35.87 ± 30.77	.400
Luminal A	6	9.33 ± 1.03	11.50 ± 15.73	.742
Luminal B	58	37.64 ± 18.44	25.57 ± 25.21	<.001
HER2 enriched	11	42.27 ± 23.38	66.82 ± 19.53	<.001
TNBC	16	50.31 ± 24.93	61.06 ± 30.05	.323

HER2 = human epidermal growth factor receptor 2, NAC = neoadjuvant chemotherapy, pCR = pathological complete response, TNBC = triple-negative breast cancer.

**Figure 7.**
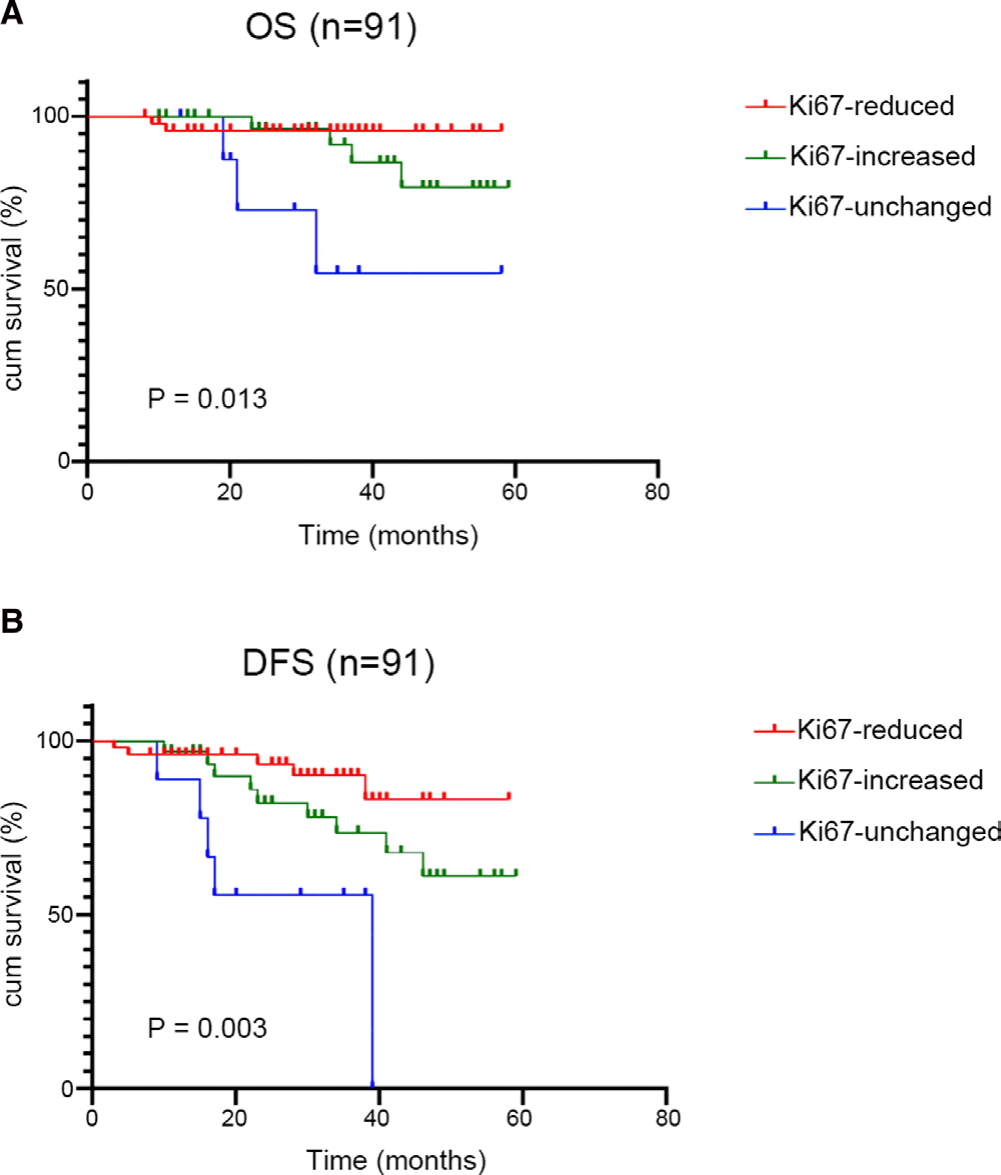
KM survival curves for OS (A) and DFS (B) of the expression of Ki67-reduced, Ki67-increased and Ki67-unchanged patients after NAC. DFS = disease-free survival, KM = Kaplan-Meier, NAC = Neoadjuvant Chemotherapy, OS = overall survival.

The OS and DFS rates of patients with Ki67 reduction after NAC were respectively 96% and 90%, and in the Ki67-increased group were 87.5% and 71.9%, respectively. In the Ki67-unchanged group, the OS and DFS rates were 66.7% and 44.4%, respectively. The survival curves are shown in Figure 7, the OS and DFS of the Ki67-reduced and the Ki67-increased group were significantly longer than the Ki67-unchanged groups (*P* = .013; *P* = .003, respectively), among which the prognosis of the Ki67-reduced patients was best.

## 4. Discussion

NAC is an approach used for patients undergoing chemotherapy before surgery, with the goal of reducing the size of a locally advanced and inoperable tumor to a size that can be surgically removed. This approach also increases the likelihood of being able to perform breast-conserving surgery. NAC has become a standard treatment for LABC.^[9,10]^ It is well-known that pCR is an ideal therapeutic effect of NAC, and previous studies showed that patients who achieved pCR after NAC might have a better prognosis.^[11,12]^ However, there remain a large number of patients who fail to respond to NAC with a risk of disease progression. Hence, it is essential for us to predict the response to NAC, as this can help identify patients who are most likely to benefit from this treatment approach.

In our current study, we evaluated the clinicopathological parameters to predict the response to NAC in breast cancer patients. The results of the univariate analysis indicated a significant correlation between patients who achieved pCR and the negative expression of the IHC markers ER and PR. Multiple clinical trials have shown that the negative status of ER and PR are favorable predictive factors for pCR of BC,^[25–27]^ and we drew a similar conclusion in this study. Additionally, in line with prior research,^[28,29]^ our study found that the pCR rate was notably higher in TNBC or HER2 enriched LABC patients compared to those with luminal A or luminal B subtypes. This suggests that patients with HR-negative expression may derive greater benefit from NAC. It is noteworthy that although previous studies suggested that HR-negative patients tend to benefit from NAC, a study by Lisa et al^[27]^ also concurrently pointed out that HR-positive BC patients tend to have a better prognosis and lower recurrence rate because of the use of aromatase inhibitors or selective ER modulators, such as Tamoxifen. Additionally, in our study, the result of the multivariate analysis showed that ER and PR were not independent indicators for predicting pCR, indicating that it remains limited to predicting the response to NAC relying solely on ER or PR. As for the cell proliferation IHC marker Ki67, previous have shown that it could serve as a predictive marker associated with pCR.^[30,31]^ However, in our research, we did not identify Ki67 as a marker associated with pCR, although the pCR rate of Ki67-high-expression patients was higher than Ki67-low-expression patients (28.1% vs 8.3%, *P* = .139), mainly due to selection bias induced by that Ki67-high-expression patients accounted for up to 90.5% of all patients.

Previous reports have indicated that HER2-positive expression is linked to a higher likelihood of achieving pCR following NAC.^[25,32,33]^ Our study corroborates these findings, as we observed a significantly higher pCR rate in HER2-positive patients compared to HER2-negative patients. Furthermore, we identified HER2 status as an independent indicator for predicting pCR in LABC patients undergoing NAC. Moreover, we further identified HER2 as an independent indicator for predicting pCR. Multiple trials and research have proven that NAC combined with trastuzumab would increase the pCR rate for HER2-positive patients.^[34]^ The NOAH trial investigated the curative effects of 1-year NAC in 235 HER2-positive LABC or inflammatory BC patients after dividing them into 2 groups randomly, with 1 group treated with only NAC and another combined trastuzumab. The result of this trial showed that the pCR rate increased from 19% to 38% with the addition of trastuzumab.^[35]^ In addition, a meta-analysis conducted by Wu et al^[36]^ included 232 HER2-positive LABC patients showed that the pCR rate of the group that received NAC combined trastuzumab was significantly higher than the only received NAC group (48% vs 26%, *P* < .0001). Consistent with previous studies, our study showed that HER2-positive patients who received targeted therapy combined with NAC had a significantly higher pCR rate (78.6%) than the patients who only received NAC (32.4%) (*P* = .003). NAC combined with targeted therapy is currently considered the standard treatment for HER2-positive LABC patients.^[37]^ However, one of the limitations of this study is that we did not analyze whether HER2-positive could predict the pCR outcome of HER2-positive patients who received NAC consisting of HER2-target-free agents due to the small number of cases.

Additionally, previous studies have suggested that patients who achieve pCR after NAC may have a better prognosis, particularly in the case of TNBC and HER2-enriched BC.^[11,12]^ In the current study, none of the luminal A patients achieved pCR, and the OS and DFS rates of TNBC, HER2 enriched, and luminal B patients who achieved pCR were higher than those of non-pCR patients. However, there was a statistically significant difference only in DFS of TNBC patients, mainly due to the small number of cases and short follow-up time of various molecular subtypes of BC. In addition, we also found that patients who achieved both tumor and ALN pCR had significantly longer DFS than both tumor and ALN non-pCR patients, suggesting that the pathological status of the ALN should also be considered as preoperative evaluation indicator to NAC for patients with ALN metastasis that might help to assess chemotherapy efficacy and prognosis.

The conversion of IHC markers after NAC has been a recent clinical research focus. Previous studies have indicated that the IHC markers of BC patients show varying degrees of alterations after NAC.^[38,39]^ In the present study, among the 91 non-pCR patients, the conversion rates of ER and HER2 were 4.4% and 6.6%, respectively. The conversion rate of PR was 22%, higher than ER and HER2, mainly changed in luminal B patients. Moreover, we found that the conversion status of HR was mainly changed from positive status to negative status, accounting for 70.8% of 24 HR conversion cases, consistent with a prospective study conducted by Jin et al^[19]^ In addition, a phenomenon was found in more than half of luminal B BC patients by Sebastian et al^[40]^ that Ki67 expression by more than 50% has reduced to less than 15% after NAC. In our study we drew a similar conclusion that Ki67 decreased expression was mainly found in the luminal B subtype, suggesting that NAC might inhibit cell proliferation in HR-positive BC. However, we did not find Ki67 decreased expression in the luminal A subtype, and we also found that Ki67 increased expression in HER-2 enriched patients, suggesting that HER2-positive expression might affect cancer cell proliferation caused by NAC. In our study, we also found that patients whose Ki67 expression decreased after NAC had longer OS and DFS than the patients without a decrease in Ki67, which is consistent with a previous study of Cabrera-Galeana et al,^[41]^ suggesting that Ki67 reduction after NAC would be a marker to predict a better prognosis for BC patients. It is well known that Ki67 is a proliferative marker thus it is not hard to understand the reduction of which after NAC reflects a lower proliferation status of cancer cells and is related to a better prognosis.^[42,43]^ Interestingly, we found that the prognosis of patients whose Ki67 increased after NAC was better than the unchanged patients, which seemed to be contradictory. However, cancer cells with a high level of proliferation status tend to be more sensitive to chemotherapy drugs thus patients would receive a better chemotherapy response and prognosis.^[44]^ No matter the increase or decrease in expression after NAC, the change of Ki67 is precisely a result of a successful therapy response. This might explain why patients whose Ki67 changed after NAC with a better prognosis than those unchanged.

The potential reasons for the conversion of IHC marker status after NAC are multifaceted. The conversion induced by NAC is primarily attributed to the varying chemosensitivity of cancer cells. Chemotherapy drugs tend to eliminate sensitive tumor cells, allowing insensitive tumor cells to proliferate and subsequently leading to the conversion of IHC markers. In addition, chemotherapy in premenopausal women might cause ovarian insufficiency to downregulate the expression of hormones and further induce HR conversion.^[45]^

However, there are still some limitations to this study. Firstly, the small number of cases might cause potential bias that many clinicopathological parameters related to pCR with differences but without statistically significant differences. Secondly, the short follow-up time, lack of recurrence, and distant metastasis cases resulted in some survival curves without statistically significant differences, despite the survival rate exist differences among different groups. Thirdly, all patients included in the study were mainly from a single region. Therefore, the conclusions we drew from this study might not be suitable for BC patients from other regions, which need further large-scale validation.

## 5. Conclusion

All in all, our study demonstrated that BC patients with negative HR status or positive HER2 status were more likely to achieve pCR after NAC. HER2 was an independent indicator for predicting pCR, and HE2-positive patients who received NAC combined targeted therapy were more likely to achieve pCR. In the present study, we observed that IHC markers of BC patients exhibit varying degrees of alterations after NAC. Patients whose Ki67 expression changed after NAC had longer OS and DFS, suggesting that Ki67 change after NAC would be a marker to predict a better prognosis for BC patients. However, these conclusions need to further be tested in large-scale populations, and the molecular mechanism of IHC conversion after NAC still needs to be further explored.

## Acknowledgments

We would like to give many thanks to our physicians, engineers, and nurses as well as other staff in the department for their extensive support.

## Author contributions

**Conceptualization:** Jundong Wu.

**Data curation:** Yutong Fang, Qunchen Zhang.

**Methodology:** Yutong Fang, Qunchen Zhang, Yuan Wu.

**Supervision:** Jundong Wu.

**Validation:** Qunchen Zhang.

**Writing – original draft:** Yutong Fang, Qunchen Zhang.

**Writing – review & editing:** Yuan Wu, Jundong Wu.
